# Experimental Modeling of Myeloproliferative Neoplasms

**DOI:** 10.3390/genes10100813

**Published:** 2019-10-15

**Authors:** Lucie Lanikova, Olga Babosova, Josef T. Prchal

**Affiliations:** 1 Laboratory of Cell and Developmental Biology, Institute of Molecular Genetics of the Czech Academy of Sciences, Videnska 1083, 142 20 Prague 4, Czech Republic; 2 Division of Hematology & Hematologic Malignancies, Department of Internal Medicine, University of Utah, School of Medicine and VAH, Salt Lake City, UT 84132, USA

**Keywords:** MPN (myeloproliferative neoplasms), zebrafish, mice, iPSCs, *JAK2*, *MPL*, *CALR*, thrombosis

## Abstract

Myeloproliferative neoplasms (MPN) are genetically very complex and heterogeneous diseases in which the acquisition of a somatic driver mutation triggers three main myeloid cytokine receptors, and phenotypically expresses as polycythemia vera (PV), essential thrombocytosis (ET), and primary myelofibrosis (PMF). The course of the diseases may be influenced by germline predispositions, modifying mutations, their order of acquisition and environmental factors such as aging and inflammation. Deciphering these contributory elements, their mutual interrelationships, and their contribution to MPN pathogenesis brings important insights into the diseases. Animal models (mainly mouse and zebrafish) have already significantly contributed to understanding the role of several acquired and germline mutations in MPN oncogenic signaling. Novel technologies such as induced pluripotent stem cells (iPSCs) and precise genome editing (using CRISPR/*Cas9*) contribute to the emerging understanding of MPN pathogenesis and clonal architecture, and form a convenient platform for evaluating drug efficacy. In this overview, the genetic landscape of MPN is briefly described, with an attempt to cover the main discoveries of the last 15 years. Mouse and zebrafish models of the driver mutations are discussed and followed by a review of recent progress in modeling MPN with patient-derived iPSCs and CRISPR/*Cas9* gene editing.

## 1. Introduction

Philadelphia chromosome-negative classical myeloproliferative disorders (more recently coined as neoplasms, MPN) are represented by polycythemia vera (PV), essential thrombocytosis (ET), and primary myelofibrosis (PMF). They are characterized by hyperplasia of at least one myeloid lineage in the bone marrow and an increased number of mature and entirely functional erythrocytes, platelets, or leukocytes, as popularized by Dameshek in the early 50s [1]. MPNs arise from a single somatically mutated hematopoietic stem cell (HSC), and the expansion of the mutated clone is accompanied by hyperplasia of a single phenotype-defining lineage. A high hemoglobin (Hb%) constitutes PV, and normal Hb% and high platelets constitute ET; however, PV patients often have elevated platelet counts, and both PV and ET may also have an elevated leukocyte count. In PMF, typical findings are anemia, neutrophilia, and thrombocytosis, or in a minority, thrombocytopenia and leukopenia, splenomegaly, immature granulocytes, increased clusters of differentiation 34+ cells (CD^34+^), nucleated red cells, teardrop-shaped red cells (dacrocytes) in the blood, marrow fibrosis, and often osteosclerosis. The high rate of proliferation is driven by the so-called ‘driver mutation’ in genes that are important for normal myeloproliferation, Janus kinase 2 (*JAK2*), and thrombopoietin receptor (*C-MPL, MPL*), or in ET and PMF, mutated calreticulin gene (*CALR*), which has acquired a novel *MPL*-activating function [2,3]. The fact that the clonal architecture, microenvironment, and mutational profile change in a given patient over time results in different phenotypes, supports the idea that MPNs are not distinct biological entities but rather a continuum in which ET transforms to PV, or chronic phase PV and ET transform to PMF, and all three transform to acute leukemia [4].

In addition to the driver mutations, loss-of-function or neomorph mutations in genes that code for epigenetic regulators, and that are shared with myelodysplastic syndromes (MDS) and acute myeloid leukemia (AML), can act as disease modifiers in MPN [5]. Besides somatic mutations, other factors such as germline variants can modulate the risk of MPN development, favor the acquisition of somatic mutations, and influence the clinical course of the disease. Furthermore, several germline mutations have been described in hereditary erythrocytosis and hereditary thrombocytosis, benign conditions represented by polyclonal hematopoiesis that, clinically, can mimic MPN and pose a difficulty in diagnosis and therapeutic management [6].

## 2. Mutational Landscape of MPN

Precise diagnosis of MPN is often challenging and has been shown to occur years after the initiation of the disease (5–10–15 years) [7]. It has been proposed that about 95–98% of PV patients carry a mutation in the *JAK2* gene, with an occurrence in ET patients of about 60% and in PMF patients of about 55% [8]. Somatic mutation *MPL* W515 occurs in 3–8% of patients with ET and PMF. Mutations in *CALR* occur in 20–35% patients with ET and PMF [3]. Noticeably, the activation of thrombopoietin (TPO) receptor (TPOR) leads to a phenotype of ET and PMF, not the PV phenotype. There are also MPN patients who do not carry any of the aforementioned mutations, so-called ‘triple-negative’ MPN patients. Triple-negative patients either carry a mutation that is as yet unknown or remains to be elucidated, or are influenced by another factor affecting their HSCs and progenitors. In fact, it has been shown that acquiring a somatic driver mutation is rather a late event in the disease process, and that other factors, such as chronic inflammation, can predispose patients’ cells to MPN transmission [9,10,11]. Additionally, polymorphisms in genes involved in DNA damage response and in the JAK/STAT pathway may increase the risk of MPN development. This includes the polymorphisms in the *JAK2* gene known as the *JAK2* 46/1 haplotype. The 46/1 haplotype was discovered by a genome-wide association study, and is a 280 Kb-long region of chromosome 9p that includes three genes in their entirety: the *JAK2* gene, insulin like 4 (*INSL4*), and insulin like 6 (*INSL6*). Surprisingly, *INSL4* and *INSL6* genes are not expressed in hematopoietic cells. There seems to be a strong association between the 46/1 haplotype and the occurrence of the *JAK2* V617F mutation; however, the precise mechanism remains to be elucidated [2,12,13,14]. A subset of patients carrying the *JAK2* 46/1 haplotype may also be predisposed to homologous recombinations of *JAK2*, followed or not by a mutation in the *JAK2* gene on the recombined allele [15].

The most frequently occurring gain-of-function *JAK2* V617F mutation gives rise to a constitutively active JAK2 kinase, which drives the JAK/STAT signaling that leads to excessive proliferation and survival of myeloid progenitor cells and accounts for >95% of driver mutations in PV and >55% in ET and PMF. Exon 12 of the *JAK2* gene is a less-frequent PV driver mutation (about 1%). Other *JAK2* mutations contributing to the MPN phenotype are under investigation [16,17]. These mutations lead to the PV phenotype and include non-synonymous substitutions, deletions and duplications, all affecting a region adjacent to the pseudokinase domain located between F533 and F547 [4,18]. The germline *JAK2* mutations were identified both in the pseudokinase (V617I, R564Q S755R) and in the kinase (R867Q, R938Q) domain [19,20,21], giving rise to the thrombocytosis phenotype. In some cases, the germline *JAK2* mutations were found to co-exist with *JAK2* V617F, further enhancing its signaling and likely predisposing the progenitor cells to the acquisition of *JAK2* V617F [22,23]. Further, two germline *JAK2* mutations, E846D and R1063H, were described in a case of hereditary erythrocytosis accompanied by megakaryocytic atypia [24], with R1063H being initially described in three out of 93 PV patients that were positive for *JAK2* V617F [17]. 

Mutations in the *JAK2* gene have been found to occur in all the cells of the hematopoietic tree starting from the HSC population, including not only a myeloid but also a lymphoid lineage [25]. Several studies point to the fact that *JAK2* V617F does not provide the HSC population with a proliferation advantage [26,27,28,29]. Patient *JAK2*-mutant xenografts in immunodeficient animal models suggest that *JAK2* mutations do not result in a self-renewal advantage. Instead, rather than enhanced self-renewal, *JAK2* V617F-positive cells expand at the progenitor level. These observations suggest that the *JAK2* V617F mutation alone is not sufficient to initiate MPN diseases, and that additional factors are required [30,31]. This is consistent with the fact that *JAK2* V617F mutation occurs in the normal population [32,33], accounting for the so-called entity of clonal hematopoiesis of indeterminate potential (CHIP) [34,35]. Intriguingly, these individuals bearing *JAK2* V617F and other CHIP somatic mutations, typically at a very low allelic burden, have increased risk of cardiovascular disease and some (but not an inevitable) risk of MPN progression. An alternative plausible explanation would be that the expansion of the progenitor pool, rather than the stem cell pool, is sufficient to induce the pathogenesis of MPN when driven by the *JAK2* mutation. This theory is supported by studies of native clonal hematopoiesis showing that a pool of long-term multipotent progenitors are the main drivers of adult hematopoiesis [36].

Activating mutations in the myeloproliferative leukemia virus (*MPL*) gene, encoding TPOR, can be either germline, such as in rare cases of familial essential thrombocytosis (*MPL* S505N) [37], or somatic. Surprisingly, the *MPL* S505N mutation has also been reported to be acquired in some rare cases of ET [38]. The most frequent somatic mutation in *MPL* is a mutation of the tryptophan residue at the 515 position (*MPL* W515) [39,40]. The mechanism by which these mutations alter TPOR signaling lies in modifying the geometry of the TPOR dimers, thus leading to transphosphorylation of the pre-bound JAK2 proteins. This results in constitutively active JAK2/STAT signaling initiated through TPOR [41,42].

Calreticulin is a multifunctional protein. It plays a role in calcium homeostasis as it binds calcium ions, rendering them inactive. Calreticulin also serves as a chaperone in the endoplasmic reticulum. However, the ET and PMF *CALR* mutations (more than 50 have been described) are all insertions or deletions that lead to frameshift mutations, resulting in their different 3’protein tails (an entirely different peptide downstream from *CALR* mutations) that acquire unique properties. This new C-terminal sequence is rich in positively charged amino acids and, unlike unmutated *CALR* (which is located in the cytoplasm), these unique *CALR*-mutated peptides are transported to the cellular membrane and activate thrombopoietin receptor. They are even secreted and activate non-mutated cells, thus acting as the *roque* cytokines [43,44].

There are other acquired mutations often reported in MPN patients. These are not restricted to MPN and frequently also occur in other hematological malignancies. They do not directly drive the clonal proliferation; nevertheless, they influence the course and progression of the disease and thus contribute to the heterogeneity of MPN. Among the most frequently reported are mutations in epigenetic regulators, splicing factors, and transcription factors, such as the tumor protein 53 (*TP53*). Out of these, the most frequently mutated are epigenetic regulators TET methylcytosine dioxygenase 2 (*TET2*) and DNA (cytosine-5)-methyltransferase 3A (*DNMT3A*). Mutations in epigenetic regulators such as enhancer of zeste homolog 2 (*EZH2*), additional sex combs like 1 (*ASXL1*), and a splicing factor, arginine/serine-rich 2 (*SRSF2*), are associated with poor prognosis and risk of AML transformation [2].

As the *JAK2* V617F mutation can drive the pathogenesis of all three classical MPNs, the question arises of how the progression of the disease differs in persons with the same mutation. A correlation between the level of expression and the phenotype has been found, with low expression being associated with an ET-like phenotype, and higher expression with a PV-like phenotype [2]. This is supported by the fact that *JAK2* exon 12 mutations exclusively lead to the PV phenotype and have been shown to activate STAT5 signaling to a greater extent [45]. Secondly, uniparental disomy (UPD) of chromosome 9 giving rise to *JAK2* V617F homozygosity is more likely associated with PV and PMF, and only rarely with ET [46,47]. This theory is also supported by knock-in mouse models, in which the ratio of mutant to wild-type Jak2 correlates with the degree of erythrocytosis [30,48]. This is also replicated in vivo in ET, when the patients that show greater *JAK2* V617F allele burden have a higher degree of erythrocytosis and leukocytosis [49].

*JAK2* V617F binds to, and stimulates, all three receptors involved in the pathogenesis of MPN erythropoietin receptor (EPOR), TPOR and granulocyte-colony stimulating factor receptor (G-CSFR). In cases of familial MPN exhibiting hereditary thrombocytosis and triple-negative MPN, it is proposed that the inherited *JAK2* mutations signal through TPOR rather than EPOR [20,21]. Differential signaling of STATs might induce differential clinical phenotypes, such as thrombocytosis being induced by TPOR/STAT1 signaling and erythrocytosis by EPOR/STAT5 [2,21,50,51]. The acquisition of somatic mutations in disease modifiers also influences the course of the disease. Further, the order of the mutation acquisition matters. It was shown that prior mutation of *TET2* altered the transcriptional program activated by *JAK2* V617F in a cell-intrinsic manner and induced the ET phenotype. In contrast, patients in whom the *JAK2* V617F was acquired first more likely present PV [52]. 

## 3. Experimental Models of MPN

In addition to the clinical data, mutational landscape exploration in patients’ samples, and in vitro experiments with cell lines simulating the impact of known alterations on hematopoietic signaling pathways, several experimental models were also created to unravel the myeloproliferative diseases’ mechanisms and dynamics. In brief, among all animal models, zebrafish have offered unsurpassed tools for in vivo functional testing of genetic variants at the cell and organism level. Zebrafish (*Danio rerio*) have been used as a model organism to study vertebrate hematopoiesis during the past two decades. They display many appealing features—easy manipulation with transparent embryos and the capacity to carry out large-scale genetic and chemical screens, allowing convenient genetic manipulation and in vivo imaging of normal and aberrant hematopoiesis [53]. The majority of hematological malignancies modeled by zebrafish represent lymphoblastic and myeloid leukemias where the transgenic lines express oncogenic fusion genes and mutations commonly found in patients [54]. MPN modeling in zebrafish introduced *jak2a* V581F (an ortholog of human *JAK2* V617F), which shared features with human PV [55]. Meanwhile, zebrafish expressing *calr* mutants have developed mpl-dependent thrombocytosis [56], and a subset of zebrafish with disrupted *asxl* genes increased their numbers of myelomonocytes [57]. These models illustrate that the signaling machinery related to the MPN phenotype is conserved between human and zebrafish and has a great potential to uncover the unique mechanisms underlying MPN.

Several mouse models have been created to characterize the role of aberrant *Jak2* signaling within the hematopoietic compartment. Retroviral transduction models, transgenic and knock-in mice bearing *Jak2* V617F concomitantly with epigenetic modifier mutations were used to study MPN maintenance and progression (reviewed elsewhere in detail [58]). The early retroviral transduction models [59,60,61,62] confirmed the role of mutated Jak2 protein in MPN pathology, in which all mice developed a PV-like disease with noticeable erythrocytosis, leukocytosis, and splenomegaly, demonstrating that the *Jak2* V617F mutation is sufficient to induce an MPN-like condition in mice. The more advanced transgenic mouse models allowing quantitative expression of *Jak2* V617F [48,63,64] indicated a correlation between the mutant Jak2 protein expression levels and MPN phenotypes progressing from ET to PV and PMF with increasing *Jak2* V617F allelic burden, thus emulating the continuous progression in MPN sub-types. In 2010, four independent groups recreated *Jak2* V617F expression in the bone marrow compartment through knock-in models with Cre-mediated recombination under the control of a specific hematopoietic promoter [58]. These mice allowed the impact of *Jak2* mutation to be studied in its endogenous environment with the native expression ratio. Once again, all studies confirmed the pivotal role of the *Jak2* V617F mutation in the onset of MPN disease and recapitulated earlier observation that the *Jak2* allele burden might affect the disease phenotype. Interestingly, all models developed a severe PV-like disorder that later progressed to myelofibrosis. Only the model by Li et al. using a human *JAK2* V617F cDNA construct under the control of endogenous murine *Jak2* promoter showed a modest ET-like phenotype, as in human ET, with only 10% of mice developing a PV-like disease, and only after 26 weeks, with marked erythrocytosis or bone marrow fibrosis [65]. Considering the crucial role of erythropoietin signaling in development of the disease [66,67], it is possible that the extensive polycythemia phenotype in these models (more severe than expected from patient studies) might reflect stronger signaling triggered by the murine EpoR when compared to human EPOR [68,69]. Double-mutant mouse models of *Jak2* V617F and epigenetic regulator *Tet2* loss-of-function, xenotransplantation-based models [70], and models using fetal liver cells expressing one or both alleles transplanted into lethally irradiated recipients [71], developed an aggressive MPN-like phenotype with rapid progression to myelofibrosis, and exhibited decreased overall survival. Detailed analysis of double-mutant early stem cells (so-called LSK, Lin^−^Sca1^+^cKit^+^) showed a strong competitive advantage over wild-type cells, suggesting that mutated *Tet2* cooperates with *Jak2* V617F in vivo to promote stem cell self-renewal and proliferation while enhancing production of late-stage stem/progenitors and resulting in disease progression through combinatorial effects [70,71]. Similar to *Tet2*, mouse models combining the loss-of-function mutation of *Ezh2* with *Jak2* V617F developed an aggressive MPN-like phenotype with an overall expansion of the LSK stem/progenitor compartment [58].

Thrombotic events are very frequent and significantly contribute to morbidity and mortality in patients with MPN, mainly PV and ET [72]. However, the pathological processes associated with thromboembolic complications in these patients are not completely understood. Several experimental evidences suggest that *JAK2* V617F-associated abnormalities in erythrocytes, leukocytes, and platelets, as well as dysfunctions of endothelial cells, might play a role [72,73]. It was shown that *Jak2* V617F mice had increased atherosclerosis caused by cellular defects in erythrocytes and macrophages, leading to increased erythrophagocytosis but defective efferocytosis [74]. Zhao et al. identified the important role of pleckstrin-2 (*Plek2*) in erythroid cell survival and enucleation during terminal erythroid differentiation [75]. By crossing *Plek2*-knock-out mice with *Jak2* V617F-knock-in mice, they were able to ameliorate the myeloproliferative phenotype and additionally rescue *Jak2* V617F-induced widespread vascular occlusion and lethality in mice [76]. *Jak2* V617F-driven MPN mouse models have also increased neutrophil extracellular trap formation, which promotes the pro-thrombotic phenotype [77]. The pathologic thrombus formation in a thrombosis model using *Jak2* V617F mice was also suppressed by blocking β1 and β2 integrin activity [78]. Remarkably, *JAK2* V617F mutation can be present not only in blood cells, but also in endothelial cells of *JAK2* V617F-positive MPN patients [79,80]. Mouse models that allow the expression of *Jak2* V617F only in endothelial cells have shown that vascular endothelial cell expression of *Jak2* V617F is sufficient to promote a pro-thrombotic state. Furthermore, treatment with hydroxyurea has reduced thrombosis and decreased the pathological interaction between leukocytes and *Jak2* V617F-expressing endothelial cells through direct reduction of endothelial P-selectin expression [81]. Overall, these findings have identified new key players in blood clotting activation in MPNs, and suggest new therapeutic targets (e.g., *Plek2*, β1/β2 integrins) and applications (e.g., achieving lower levels of hematocrit in patients due to the direct role of *JAK2* V617F-positive erythrocytes in promoting advanced atherosclerosis [74]).

The pathogenic role of increased ectopic expression of murine *Tpo* receptor [82] or high thrombopoietin production by hematopoietic cells [83] were known long before *MPL* mutations were discovered in MPN patients [38,39]. In 2006, the retroviral transduction mouse model generated by Pikman et al. [39] expressing the most common *Mpl* mutation, W515L, resulted in a fully penetrant myeloproliferative disorder characterized by marked thrombocytosis, which increased reticulin fibrosis and induced splenomegaly due to extramedullary hematopoiesis. Importantly, the effect of *Mpl* W515L was lost when *Jak2* was deleted from recipient cells (Mx-Cre-Jak2^flox/flox^ knock-out cells) [84], and *Jak2* V617F/Mpl^−/−^ transgenic mice [85] exhibited reduced thrombocythemia, neutrophilia, splenomegaly, and neoplastic stem cell pools. This suggests that *Mpl* expression, but not TPO, has a fundamental effect on MPN development and severity. In addition, these results have demonstrated that the *Mpl* W515L clone relies on the presence of wild-type Jak2 kinase to maintain the MPN phenotype and vice versa, that wild-type *Mpl* is indispensable in the development of *Jak2* V617F MPN-like diseases in mice. The mouse models with mutated *Calr* cover the two most commonly seen mutations in humans, *CALR*del52 and *CALR*ins5 [86], and are consistent with the ET phenotype in humans—all the models displayed isolated thrombocytosis with no significant effect on erythrocyte or leukocyte counts [58]. 

In conclusion, mouse models are a useful tool for studying the impact of the most prominent mutations on progression of MPN diseases. Since there are only limited data available describing the biochemical compatibility of human and mouse cytokines and their interaction with appropriate receptors (EPOR, MPL, or G-CSFR), it is important, particularly in murine models and derived cell lines, to co-express same-species receptors and use same-species cytokines, or to appropriately describe and discuss the experimental set-up. In addition, advances in understanding the critical role of JAK2/STAT signaling have allowed the design of potential MPN therapies, such as JAK2 inhibitors and murine models, which have been proven to be suitable candidates for screening and testing new promising compounds, although these do not fully allow the MPN heterogeneity seen in human patients to be addressed. 

The derivation of human-induced pluripotent stem cells (iPSCs) in 2007 [87] began a new era in the modeling of human diseases, and introduced the generation of disease- and clone-specific iPSC lines that preserve the genetic identity of hematopoietic stem/progenitor clones. The first MPN-specific iPSCs were derived from CD^34+^ cells isolated from the peripheral blood of both PV and PMF patients with heterozygous *JAK2* V617F mutations and an allele burden of approximately 50% [88]. The reprogramming protocol was based on Yamanaka retroviral factors (*Oct4, Sox2, Klf4* and *c-Myc*), which were transduced to pre-activated CD^34+^ cells, and in total 11 clones were expanded and characterized. The expanded *JAK2* V617F iPSC clones displayed characteristics of pluripotent human embryonic stem cells with normal karyotypes and allowed direct differentiation into hematopoietic cells (CD^34+^/CD^45+^). Further evaluation of erythroid potential identified a two-fold proliferation advantage in the *JAK2* V617F clones over the normal controls. In addition, PV-iPSC-generated hematopoietic progenitor cells showed a PV-unique gene expression pattern corresponding to the primary CD^34+^ cells. This pivotal experiment showed that, similar to human iPSCs derived from fibroblasts and normal CD^34+^ cells, MPN cells can be directly reprogrammed with efficiencies comparable to those of normal cells. In contrast, MDS and AML cells are significantly more refractory to the reprogramming [89]. While bone marrow genetic heterogeneity hampers the isolation of individual oncogenic subclones, generation of MPN-iPSCs allows reconstruction of the clonal hierarchy and investigation of the effects of the mutations in their endogenous loci. In addition, advanced genome editing techniques (e.g., CRISPR/*Cas9* technology) offer allele-specific gene targeting based on homologous donors and the generation of isogenic corrected lines. It has already been shown that the efficacy of the CRISPR/*Cas9* system in targeting the *JAK2* V617F allele in PV-iPSCs is more than 80% [90]. Whereas high-frequency off-target mutagenesis induced by CRISPR/*Cas9* nucleases have been reported in some human cells [91], the targeted deep sequencing of edited PV-iPSCs clones has revealed high specificity with only minimal off-target effects [90,92].

The attempt to decipher the pre-*JAK2* V617F predisposing genetic lesions by PV-iPSCs has identified several candidate genes that, however, await further functional characterization [93]. Isogenic human erythroblasts and hematopoietic progenitors generated from PV patient-specific iPSCs have been used to examine responses to clinically used kinase inhibitors, especially JAK2 inhibitors. Saliba et al. used inhibitors targeting different signaling pathways (INCB018424 (Ruxolitinib)—JAK2 and JAK1; TG101348 (SAR302503)—JAK2 and FLT3; Ly294002—PI3K; RAD001—mTOR and AUY922—HSP90) and showed inhibition of erythroid growth in a dose- dependent manner in all the generated cell lines, regardless of their *JAK2* status [94]. The main disadvantage of retrovirally generated iPSCs is the methylation-induced silencing of transgenes and the random integration of retroviruses, which may affect the differentiation potential of the derived iPSC lines. Non-integrational, virus-free iPSC derivation by episomal vectors can overcome these problems, and was used by Ye et al. to generate iPSCs with distinct *JAK2* V617F allele compositions from one female PV patient [95]. The authors investigated the capacity of INCB018424 (Ruxolitinib), TG101348 (SAR302503), and CYT387 to suppress mutated *JAK2* V617F in PV-iPSC-derived myeloid and erythroid cells. Similar to the previous study and to the clinical findings [96,97], all three drugs non-selectively inhibited erythropoiesis in normal and PV-iPSC lines; however, the JAK inhibitors had a lower inhibitory effect on the self-renewal of iPSC-derived CD^34+^ hematopoietic progenitors, explaining the failure to eradicate the *JAK2* V617F clones after the treatment. Generation of human iPSCs from ET and PMF patients carrying *MPL* V501L and *CALR*ins5 has been reported, but detailed analysis of their erythroid and megakaryocytic differentiation potential is still ongoing [98,99]. Transcriptomic and proteomic analyses of megakaryocyte progenitors derived from *CALR* mutants and CRISPR/*Cas9*-corrected isogenic iPSC lines are ongoing as well [100]. Overall, MPN-iPSCs recapitulate the disease phenotype in vitro, and have been proven suitable for studying MPN pathogenesis, clonal architecture, and drug efficacy.

## 4. Conclusions

MPN molecular pathogenesis has been extensively elucidated by discoveries of MPN driver and modifier mutations during the last 15 years. Thanks to the combination of in vitro studies and animal modeling, it is now clear that aberrant hematopoietic cytokine receptors/JAK2 cooperation and, consequently, abnormal signaling of their downstream partners, can replicate most of the MPN phenotypes. Nevertheless, the molecular mechanisms of the disease’s initiation are still not completely understood, and the exact role of the contribution of important disease-modifying factors such as aging, inflammation, and germline genetic predisposition await further study. Undoubtedly, *JAK2* V617F mutation is frequently present, but has insufficient penetrance to give rise to the MPN disease or three different MPN phenotypes. Therefore, it will be important in the future to model the oncogenic cooperation between MPN driver and other acquired or germline mutations with extrinsic factors and genetic abnormalities (e.g., by using a combination of CRISPR/*Cas9* and iPSCs techniques). Initial studies have already produced interesting new insights by modelling the DNA-damaging inflammatory microenvironment using induced pluripotent stem cell-derived CD^34+^ progenitor-enriched cultures from a *JAK2* V617F PV patient. It was shown that *JAK2* V617F PV progenitors utilize dual-specificity phosphatase 1 (DUSP1) activity as a protection mechanism against DNA damage accumulation, promoting their proliferation and survival in the inflammatory microenvironment [101].
